# Unraveling the Influence of K280 Acetylation on the Conformational Features of Tau Core Fragment: A Molecular Dynamics Simulation Study

**DOI:** 10.3389/fmolb.2021.801577

**Published:** 2021-12-13

**Authors:** Yu Zou, Lulu Guan

**Affiliations:** Department of Sport and Exercise Science, College of Education, Zhejiang University, Hangzhou, China

**Keywords:** tau, acetylation, oligomers, conformational features, replica exchange molecular dynamics

## Abstract

Abnormal aggregation of the microtubule-associated protein Tau is closely associated with tauopathies, including Alzheimer’s disease and chronic traumatic encephalopathy. The hexapeptide 275VQIINK280 (PHF6*), a fibril-nucleating core motif of Tau, has been shown to play a vital role in the aggregation of Tau. Mounting experiment evidence demonstrated the acetylation of a single-lysine residue K280 in the PHF6* was a critical event for the formation of pathological Tau amyloid deposits. However, the underlying mechanisms by which K280 acetylation affects Tau aggregation at the atomic level remain elusive. In this work, we performed replica exchange molecular dynamics simulations to investigate the influence of acetylation of K280 on the aggregation of PHF6*. Our simulations show that acetylation of K280 not only enhances the self-assembly capability of PHF6* peptides but also increases the β-sheet structure propensity of the PHF6*. The inter-molecular interactions among PHF6* peptides are strengthened by the acetylation of K280, resulting in an increased ordered β-sheet-rich conformations of the PHF6* assemblies along with a decrease of the structural diversity. The residue-pairwise contact frequency analysis shows that K280 acetylation increases the interactions among the hydrophobic chemical groups from PHF6* peptides, which promotes the aggregation of PHF6*. This study offers mechanistic insights into the effects of acetylation on the aggregation of PHF6*, which will be helpful for an in-depth understanding of the relationship between acetylation and Tau aggregation at the molecular level.

## Introduction

Tau, a microtubule-associated protein first discovered in 1975, was found to be critical for the assembly and stabilization of microtubules (Weingarten et al., 1975). Recent advances revealed that Tau also played an important role in a diverse range of molecular pathways including cell signalling, synaptic plasticity, and regulation of genomic stability (Brandt and Lee, 1993; Bhaskar et al., 2005; Regan et al., 2017). Tau is a highly soluble, natively unfolded protein, while it can aberrantly assemble into insoluble aggregates under disease conditions. The abnormal deposition of modified Tau is the pathognomonic hallmark of many neurodegenerative diseases called “tauopathies”, including but not limited to Alzheimer’s disease (AD), progressive supranuclear palsy (PSP), and chronic traumatic encephalopathy (CTE) (Lee et al., 2001; Arendt et al., 2016).

The physiological functions of Tau are regulated by various post-translational modifications (PTMs), including phosphorylation, acetylation, glycation, nitration, O-GlcNAcylation, oxidation, ubiquitination, SUMOylation and methylation. Approximately 35% of the amino acid residues in Tau are susceptible to modification post-translationally (Wang and Mandelkow, 2016). Many PTMs have been identified in Tau extracted from healthy brains, suggesting a normal role for PTMs in the function of Tau (Haj-Yahya and Lashuel, 2018). However, aberrant PTMs reduce the ability of Tau to bind to microtubules, and promote the pathological Tau aggregation (Alonso et al., 1994; Irwin et al., 2012). Recently, Wesseling et al. generated a high-resolution quantitative proteomics map of 95 PTMs on multiple isoforms of Tau, and demonstrated that these modifications occurred in an ordered manner and led to Tau aggregation (Wesseling et al., 2020).

Tau hyperphosphorylation is widely considered as a trigger for the formation of aggregates. Many *in vitro* and *in vivo* studies have showed the close relationship between abnormal Tau phosphorylation and aggregation (Alonso et al., 1996; Haase et al., 2004; Lyons et al., 2014; Derreumaux et al., 2020). Other modification like acetylation was reported to be as important as phosphorylation in dictating the biophysical properties of Tau. Recent study suggested that Tau acetylation was a common factor in both traumatic brain injury (TBI) and AD, and may hold promise as a therapeutic target and potential blood biomarker of tauopathies (Shin et al., 2021). Tau has 44 lysine residues (10% of all residues) and more than 20 lysine residues are subject to acetylation (Min et al., 2010). Among these acetylation sites, Tau acetylated at K174, K274, K280, and K281 have been found in the brains of patients with AD, and receive most attention concerning their significance in regulating Tau function. K174 is located in the proline-rich region of Tau, and pseudo-acetylation at this site can increase Tau accumulation and regulate Tau-induced toxicity (Min et al., 2015). Abnormal acetylation of K274 and K281 have been reported to impair Tau-mediated microtubule stabilization, and enhance the formation of Tau aggregates and the cytotoxicity of Tau oligomers (Trzeciakiewicz et al., 2017; Rane et al., 2019). This contrasts with acetylation at K174, which induces Tau aggregation without affecting Tau-microtubule binding (Min et al., 2015). Besides, *in vivo* study suggested that pathological acetylation of K274 and K281 promoted memory loss and disrupted synaptic plasticity, leading to an impaired hippocampal long-term potentiation (Tracy et al., 2016).

Acetylation of K280 is of particular interest among these four modifications since it is located within the PHF6* motif (nucleating segments for Tau assembly) of the repeat region. Acetylated K280 is detected in neuronal and glial inclusions in many tauopathies (such as AD, frontotemporal dementia with parkinsonism-17, corticobasal degeneration and PSP), but not in normal brains (Cohen et al., 2011; Irwin et al., 2012; Irwin et al., 2013). Acetylation on this site may impair the microtubule assembly and reduce solubility of Tau, leading to the losses of normal Tau properties. Moreover, K280 acetylation was reported to increase the phosphorylation at S262 and T212/S214 and enhance total Tau amounts in *Drosophila* and mice model (Cohen et al., 2011; Gorsky et al., 2016). The results of the effect of K280 acetylation on Tau aggregation, however, have been equivocal; some experimental studies have demonstrated the increased aggregation after acetylation at K280 (Cohen et al., 2011; Trzeciakiewicz et al., 2017; Haj-Yahya and Lashuel, 2018), while the decreased aggregation and the fibrillation rate of Tau were also reported (Kamah et al., 2014; Ferreon et al., 2018). Therefore, uncovering the molecular mechanism by which K280 acetylation affects Tau aggregation at the atomic level will provide a supplement for the above-mentioned experiments. In recent years, some computational studies have focused on the relationship between acetylation and Tau aggregation. By performing replica exchange molecular dynamics (REMD) simulations, Luo et al. explored the conformational ensemble of Tau K18 and K19 monomers, and found that the ordered conformations with close lysine-cysteine distances could facilitate Tau self-acetylation (Luo et al., 2014). Kim et al. evaluated the acetylation state of 27 human Tau lysine residues and concluded that 15 acetylation mimics were predicted to be detrimental, and acetylation at the site of K311 may increase Tau aggregation propensity (Kim and Jeong, 2019). Despite these observations, to our knowledge, the atomistic details of the effect of K280 acetylation on Tau aggregation have not been unveiled by simulations to date. In this study, we investigated the effect of K280 acetylation on oligomerization of the nucleation core fragment PHF6* by carrying out 400 ns all-atom explicit-solvent REMD simulations. We found that acetylation of K280 could promote the formation of β-sheet structure and enhance the aggregation propensity of PHF6*, consistent with previous experimental studies (Cohen et al., 2011; Trzeciakiewicz et al., 2017; Haj-Yahya and Lashuel, 2018). PHF6* oligomers have pronounced structural diversity, and the K280 acetylation strengthens the intermolecular interactions, resulting in a reduced morphological diversity. In addition, K280 acetylation obviously changes the hydrogen bond (H-bond) and contact maps, and increases the residue-residue interactions.

## Material and Methods

### Peptide Systems

PHF6* consists of six residues, and its amino acid sequence is ^275^VQIINK^280^. To investigate the effect of acetylation at residue K280 on the conformational ensemble of PHF6* oligomers, we simulated two different systems: the wild type PHF6* system (named PHF6* system) and the K280 acetylated system (named Ac-PHF6* system). Each system consists of twelve peptide chains with random coil conformation for each chain, which are the final conformations generated in a 50 ns molecular dynamics (MD) simulation at 450K in water starting from fully extended peptides. To eliminate the effects of terminal charges, the peptides in two systems were capped by the ACE (CH3CO) group at the N-terminus and the NH2 group at the C-terminus, as done experimentally and computationally by Levine et al. (Levine et al., 2015). The chains were placed randomly in a 6.88 × 6.88 × 6.88 nm^3^ box filled with TIP3P water molecules (Jorgensen et al., 1983). The total numbers of atoms for the two systems are 31,869 and 31,911, respectively.

### Details of REMD Simulation

All REMD simulations were performed using the Gromacs 2018.4 software package with all-atom Amber99SB-ILDN force field (Van et al., 2005; Lindorfflarsen et al., 2010; Abraham et al., 2015). There are 48 replicas for both PHF6* and Ac-PHF6* systems, each of 400 ns duration, with a temperature range of 308–414 K (Supplementary Table S1). Replica exchange was attempted every 2 ps and periodic boundary conditions were applied in all replicas. Lengths of chemical bonds within protein and water molecules were constrained respectively by the LINCS and SETTLE algorithms (Miyamoto and Kollman, 1992; Hess et al., 1997), allowing an integration time step of 2 fs. The pressure was kept at 1 bar using the Parrinello-Rahman method with a coupling time constant of 1 ps (Parrinello and Rahman, 1981). The Particle Mesh Ewald method was used to calculate the electrostatic interactions with a real space cutoff of 1.2 nm (Darden et al., 1993), and the van der Waals interactions were calculated using a cutoff of 1.2 nm.

### Analysis Methods

The data analyses were performed using tools implemented in the Gromacs package and our in-house-developed codes. We chose the last 100 ns simulation data for analyses as the first 300 ns data of each replica may have bias of the initial structures. The temperature used in data analysis in our simulations is 310 K (physiological temperature). The secondary structures of peptides were identified using the dictionary secondary structure of protein (DSSP) program (Kabsch and Sander, 1983), and the β-strand length was referred to the number of consecutive residues that adopted β-strand. The Daura analysis method was applied to classify the conformations sampled using a Cα-root-mean-square deviation (Cα-RMSD) cutoff of 0.45 nm (Daura et al., 1999). One H-bond is considered to be formed when 1) the distance between N and O is less than 0.35 nm and 2) the angle of N-H···O (or O-H···N) is larger than 150° (van der Spoel et al., 2006). The potential of mean force (PMF) was constructed using the equation −*RT* ln *H* (x, y), where *H* (x, y) is the histogram of two selected reaction coordinates, x and y. Here, x and y refer to the number of H-bonds and radius of gyration (Rg) of PHF6* oligomers, respectively. A contact is defined when two carbon atoms or a carbon atom and another heavy atom of two non-sequential residues lie within 0.54 nm, or any other two heavy atoms of two non-sequential residues come within 0.46 nm (Zou et al., 2019; Li et al., 2021).

## Results and Discussion

Before analyzing the simulation data, the convergence of the two REMD simulations was examined by comparing the following several parameters within two different time intervals (300–350 and 350–400 ns) for the PHF6* and Ac-PHF6* systems at 310 K. These parameters include the average probability of secondary structure (including coil, β-sheet, β-bridge, bend and turn) over all residues, secondary structure (coil and β-sheet) probability of each residue, the probability density function (PDF) of the number of H-bonds and Rg of PHF6* oligomers, and the end-to-end distance of each peptide chain in the oligomers. As shown in Supplementary Figure S1, Supplementary Figure S2, these five parameters within the two time intervals are quite similar for both systems, indicating that two REMD simulations are reasonably converged after 300 ns. Therefore, all the analyses presented below were performed using the converged data.

### K280 Acetylation Increases the β-Sheet Propensity of PHF6* Peptides and Promotes the Formation of Larger β-Sheet-Rich Oligomers.

**FIGURE 1:**
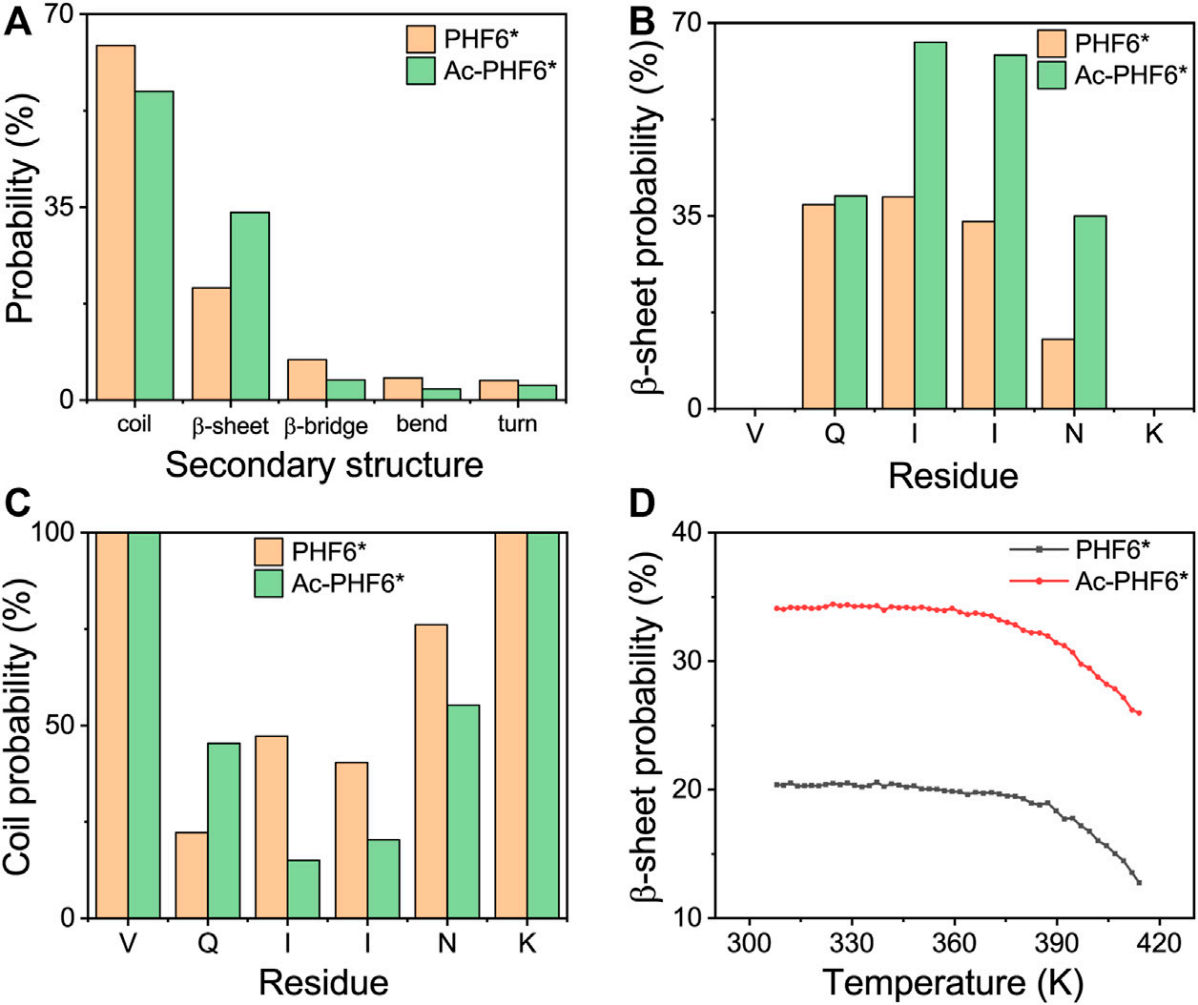
Analysis of secondary structure probability of PHF6* and Ac-PHF6* systems: average probability of each secondary structure (coil, β-sheet, β-bridge, bend and turn) over all residues **(A)**, probability of β-sheet **(B)** and coil **(C)** as a function of each amino acid residue, average probability of β-sheet **(D)** at different temperatures.

To investigate the effect of acetylation at K280 on the structural properties of PHF6* oligomers, we first calculated the secondary structure compositions (including coil, β-sheet, β-bridge, bend, and turn) over all residues, and the residue-based β-sheet and coil probability in two systems. As shown in Figure 1A, the probability of coil and β-sheet in the PHF6* system is 64.3 and 20.3%, respectively. Qi et al. performed REMD simulations on truncated Tau (K18) monomer and reported the average β-structure propensity of PHF6* is 23.9% (Qi et al., 2015), close to that (β-sheet and β-bridge propensity, 27.7%) generated in our study. When K280 is acetylated, the coil structure content decreases to 56.0% and the β-sheet content markedly increases to 34.0%. The residue-based β-sheet probability (Figure 1B) shows that the PHF6* peptides have 12.6–38.5% probabilities to adopt β-sheet states, with hydrophobic residues I277, I278, and N279 having, respectively, a probability of 38.5, 34.0 and 12.6%. After acetylation, all of the residues have an increased β-sheet probability (35.0–66.5%), with hydrophobic residues having a probability of 66.5, 64.2 and 35.0% respectively. The PHF6* system has a coil percentage of 22.0–76.1% in the above-mentioned residues, while a decreased coil percentage of 15.1–55.2% appears in the Ac-PHF6* system. We also investigated the β-sheet probability as a function of temperature in two systems in Figure 1D. In the PHF6* system, the probability of the β-sheet decreases with increasing temperature, indicating that the PHF6* peptides prefer to aggregate at lower temperatures. The similar phenomenon is also seen in other short peptides, such as Aβ_16-22_ (Xie et al., 2013), Aβ_30-36_ (Qian et al., 2017) and hIAPP_11-25_ (Qi et al., 2014). In the Ac-PHF6* system, significantly higher β-sheet probabilities are observed at all simulated temperatures, with a probability of 34.1% at 308 K and 26.0% at 414 K. These results demonstrate that K280 acetylation markedly increases the β-sheet probability and facilitates the β-sheet formation of PHF6* peptides.

**FIGURE 2:**
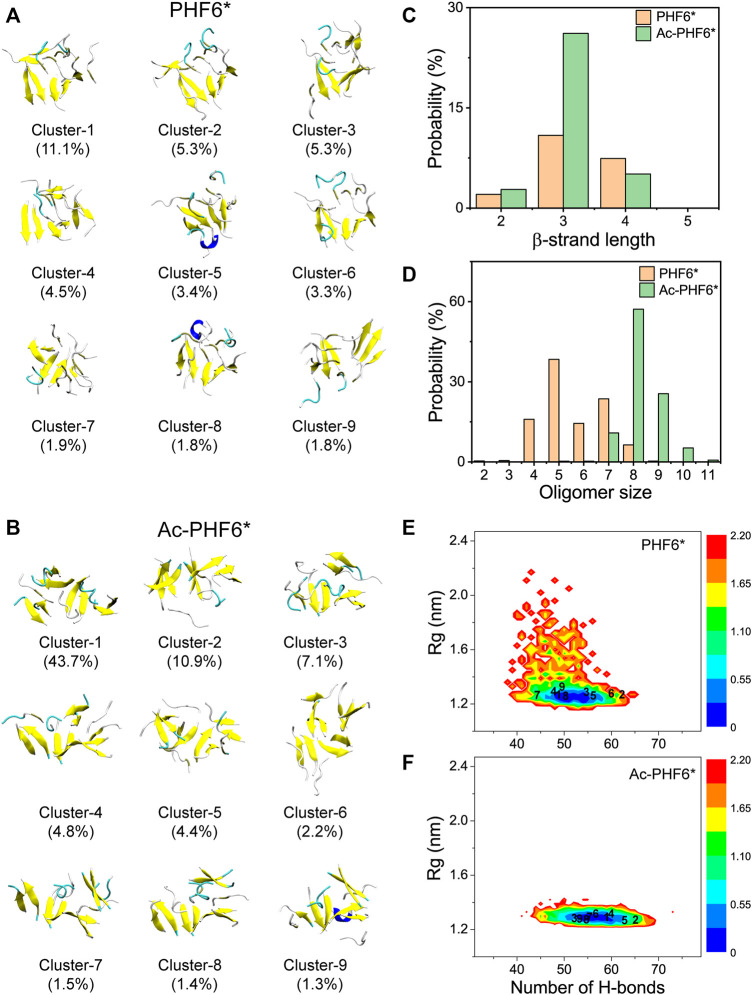
Representative conformations of the first nine most-populated clusters of PHF6* oligomers in PHF6* **(A)** and Ac-PHF6* **(B)** systems. The corresponding population of each cluster is given in parentheses. Probability of the β-strand length **(C)** and different sizes of oligomers **(D)** in two systems. The potential of mean force (PMF) (or free energy surface) (in kcal mol^−1^) of PHF6* oligomers in PHF6* **(E)** and Ac-PHF6* **(F)** systems projected on two reaction coordinates: the H-bond number and the Rg of the PHF6* oligomers. The numbers in the PMF correspond to the cluster index.

After comparing the secondary structure difference between two systems, we further performed an RMSD-based cluster analysis using a Cα-RMSD cutoff of 0.45 nm to investigate the three-dimensional (3D) conformations of PHF6* oligomers in two systems. The conformations in the PHF6* and Ac-PHF6* systems are separated into 493 and 119 clusters, respectively. The larger cluster number in PHF6* system reflects that the structural diversity of PHF6* oligomers in PHF6* system is more pronounced than that in Ac-PHF6* system. The top nine most-populated clusters and their populations are shown in Figures 2A,B, which represent 38.4 and 77.3% of all conformations in the PHF6* and Ac-PHF6* systems, respectively. The percentages of the nine clusters in Ac-PHF6* system are much higher than those in PHF6* system, again indicating the high similarity and low diversity of conformations in Ac-PHF6* system. As seen in Figure 2A, the PHF6* oligomers in the top nine clusters for the PHF6* system mainly adopt disordered β-sheet-rich conformations, with some collapsed coil aggregates. In the Ac-PHF6* system, the length of β-strand is longer than that in the Ac-PHF6* system, and more extended β-sheets and barrel-like structures (Cluster-2) are observed in these clusters. It is noted that β-barrel structure was reported to be cytotoxic (Laganowsky et al., 2012), and a number of studies (Lei et al., 2016; Zou et al., 2016; Chen et al., 2018) found that 6–10 chains of amyloid peptides (including P53_251-257_, SOD1_147-153_ and Aβ_33-42_) can form closed or open β-barrel structures. In order to examine the effects of acetylation on the formation of barrel-like structures, we calculated the probability of different sizes of β-barrel in PHF6* and Ac-PHF6* systems. As shown in Supplementary Figure S3A, in the PHF6* system, the four- and five-stranded β-barrels have relatively high probabilities of 6.9 and 3.4%, respectively. When K280 is acetylated, the populations of the four- and five-stranded β-barrels are greatly enhanced (with a probability of 11.2 and 11.9%), and larger sizes (6–8) of β-barrels appear, demonstrating that K280 acetylation can promote the formation of β-barrels and increase the β-barrel size of PHF6*. Some represented barrel-like and bilayer β-sheet structures in the Ac-PHF6* system are presented in Supplementary Figure S3B. Next, to examine whether the differences of the conformations between two systems are statistically significant, we calculated the distribution of β-strand length in the PHF6* oligomers. Figure 2C shows that in the PHF6* system, the majority of the β-strand has a length of three residues. The two-, three- and four-residue β-strand have a probability of 2.1, 10.9 and 7.4%, respectively. When K280 is acetylated, the three-residue β-strand has a significantly increased probability of 26.2%, and the four-residue β-strand displays a slightly decreased probability of 5.1%. Despite the decrease in four-residue β-strand, the total probability of long β-strands (31.3%, sum of the probabilities of three-, and four-residue β-strands) in the Ac-PHF6* system is much higher than that (18.3%) in the PHF6* system. The probability distribution of different size of oligomers is presented in Figure 2D. It can be seen that in the PHF6* system, PHF6* peptides have relatively high probability to form pentamer (38.4%), followed by heptamer (23.6%), tetramer (15.9%), hexamer (14.4%) and octamer (6.4%). In the Ac-PHF6* system, PHF6* peptides form predominantly octamer and nonamer, with a probability of 57.1 and 25.5%, respectively. Larger size oligomers are observed, and the tetramer, pentamer and hexamer vanish after acetylation. These data indicate that K280 acetylation increases the population of long β-strands, promotes the formation of larger β-sheet-rich oligomers and enhances the self-assembly capability of PHF6* peptides.

To explore the influence of acetylation on the whole conformational space of the PHF6* oligomers, we plotted the PMF (or free energy surface) as a function of two reaction coordinates, the number of H-bonds and Rg of PHF6* oligomers in two systems. The locations of the first nine clusters are labeled on the PMF plot. As seen in Figures 2E,F, the PMF in the Ac-PHF6* system is quite concentrated, while it is scattered in PHF6* system, in accordance with the different cluster numbers (493 and 119) in two systems. The H-bond number and Rg in the Ac-PHF6* system vary from 39 to 73 and 1.23 to 2.04 respectively, while those in the PHF6* system range from 32 to 66 and 1.19 to 2.90. The locations of the dominant minimum-energy basins for two systems are also very different. In the PHF6* system, the free energy surface contains two minimum-energy basins, centered at (number of H-bonds, Rg) values of (50, 1.24 nm) and (51, 1.24 nm). In the Ac-PHF6* system, only one energy basin located at the value of (56, 1.3 nm) is observed. These results indicate that K280 acetylation promotes the formation of H-bonds and induces more ordered aggregates, thus alter the whole free energy surface.

**FIGURE 3:**
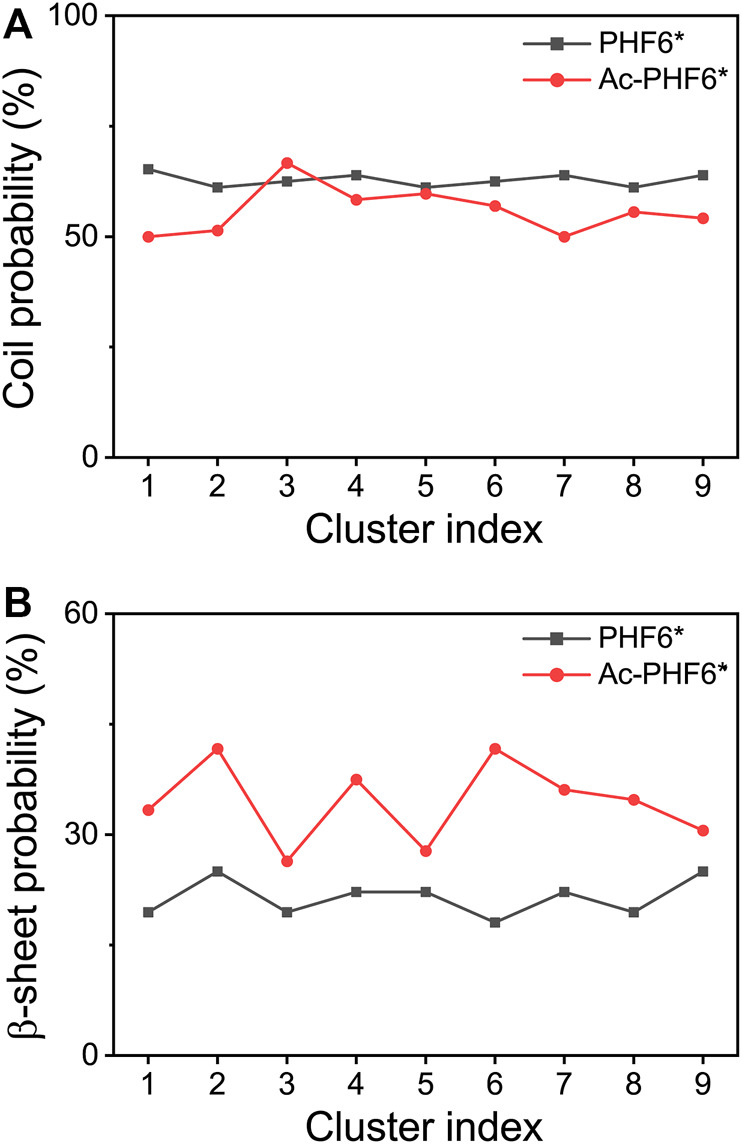
Averaged probabilities of coil **(A)** and β-sheet **(B)** as a function of cluster index in two systems. The cluster with lower index has a higher sampling probability.

We also calculated the coil and β-sheet probabilities as a function of cluster index to investigate the dominant secondary structure probability of the most-populated conformations. As seen in Figure 3, the secondary structure probability distributions are quite different in two systems. The coil probabilities in the majority of conformations are decreased after acetylation except for the Cluster-3 (with a slight increase of 4.2%). The β-sheet probabilities of the first nine most-populated conformations are all increased upon the acetylation of K280, with an average increment of 13.0%. This result further indicates K280 acetylation has the ability to accelerate the formation of β-sheet-rich structures.

### Acetylation of K280 Induces PHF6* Peptides to Form a More Compact State

**FIGURE 4:**
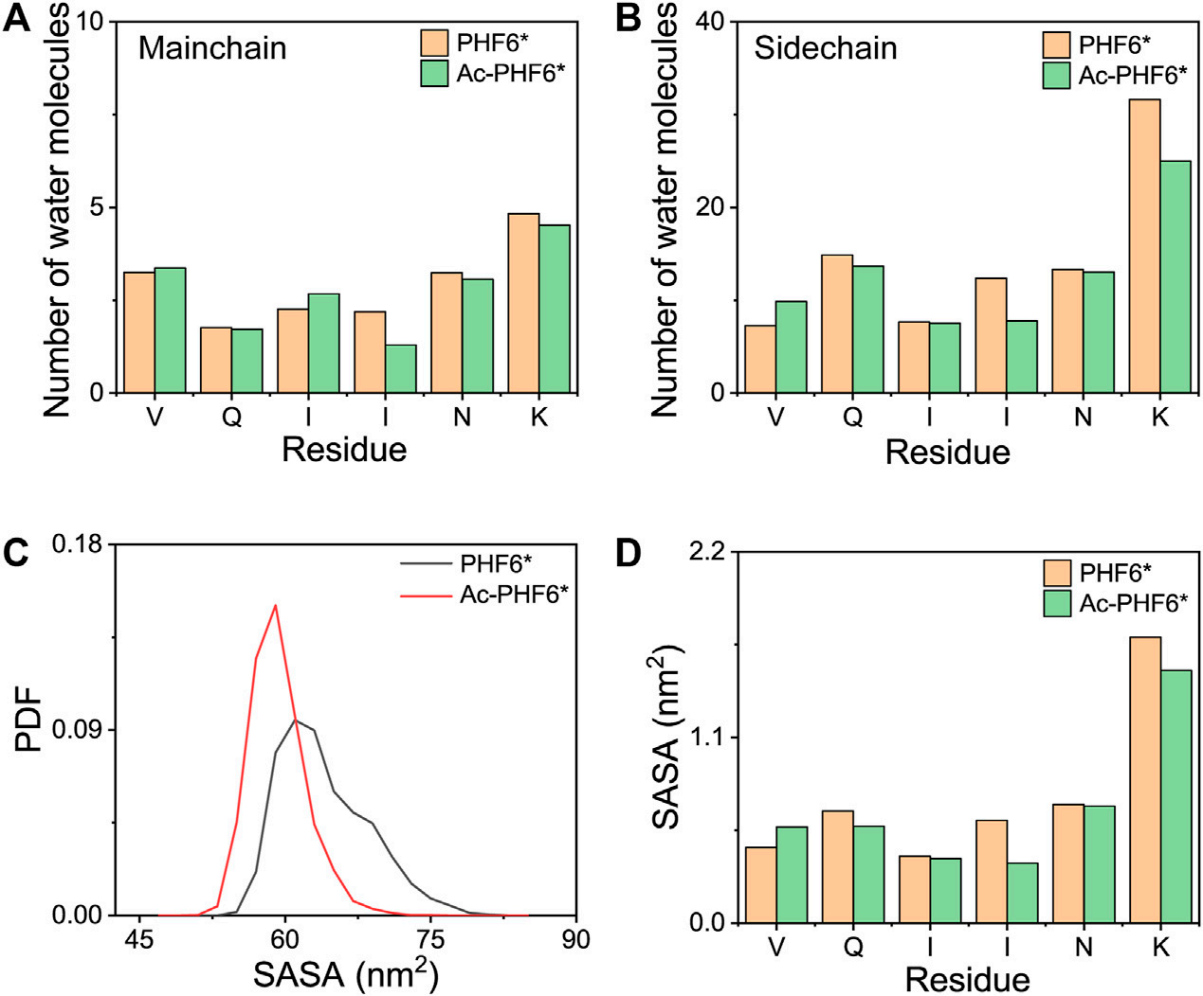
The average number of water molecules within 0.35 nm of the mainchain **(A)** and sidechain **(B)** atoms of each residue in PHF6* and Ac-PHF6* systems. Probability distribution function of the solvent accessible surface area (SASA) for all residues **(C)** and SASA as a function of each residue **(D)**.

It is interesting to monitor the extent of solvation of the peptide and look at the role of water in the REMD simulations (Krone et al., 2008). To this end, we calculated the average number of water molecules within 0.35 nm from the mainchain and sidechain atoms of each residue. As seen in Figure 4A, for the mainchain atoms, the terminal residues V275 and K280 have relatively larger number of contacting water molecules than other residues, and the three residues (QII) in the middle of the amino acid sequence of the PHF6* peptide are well protected from the solvent. Figure 4B shows that for the sidechain atoms, residue K280 is far more solvent-exposed than other residues. When comparing the number of water molecules in two systems, we found that for mainchain and sidechain atoms, the number of water molecules around the majority of the residues decreases after acetylation, indicating that K280 acetylation decreases the water exposure of peptide atoms and weakens the peptide-water interaction, thus strengthening the peptide-peptide interaction. Figures 4C,D shows the PDF of SASA for all residues and SASA as a function of each residue. The peptides in Ac-PHF6* system display a smaller SASA peak value than those in the PHF6* system, indicating that K280 acetylation induces PHF6* peptides to form a more compact state. More specifically, the SASA values of Q276, I277, I278, N279 and K280 are decreased after acetylation, showing that these residues prefer to orientate to the interior of oligomers. In addition, we also plotted the end-to-end distance probability distributions for all chains in two systems in Supplementary Figure S4. In the PHF6* system, a broad ensemble of states is observed between 0.45 and 1.75 nm (black curve), and there exist a sharp peak at a value of 1.65 nm and two smaller peaks at 0.85 and 1.15 nm, respectively. As shown in Supplementary Figure S4B, PDF peak value of 1.65 nm corresponds to the β-strand conformations, and values of 0.85 and 1.15 nm correspond to the non-β-sheet conformations. When K280 is acetylated, the ensemble shifts between 0.35 and 1.75 nm (red curve), and two smaller peaks disappear and the sharp peak at the value of 1.65 nm is much higher. These results indicate that the PHF6* peptides in the Ac-PHF6* system become more extended as the long extended β-strand formations are enhanced by K280 acetylation. The structures in the Ac-PHF6* system coexist in a balance of compact states and extended fibril-like β-sheet conformations, which are also reported in the dimeric structures of Aβ_25-35_ (Wei et al., 2010).

### K280 Acetylation Significantly Strengthens the Residue-Residue Interactions

**FIGURE 5:**
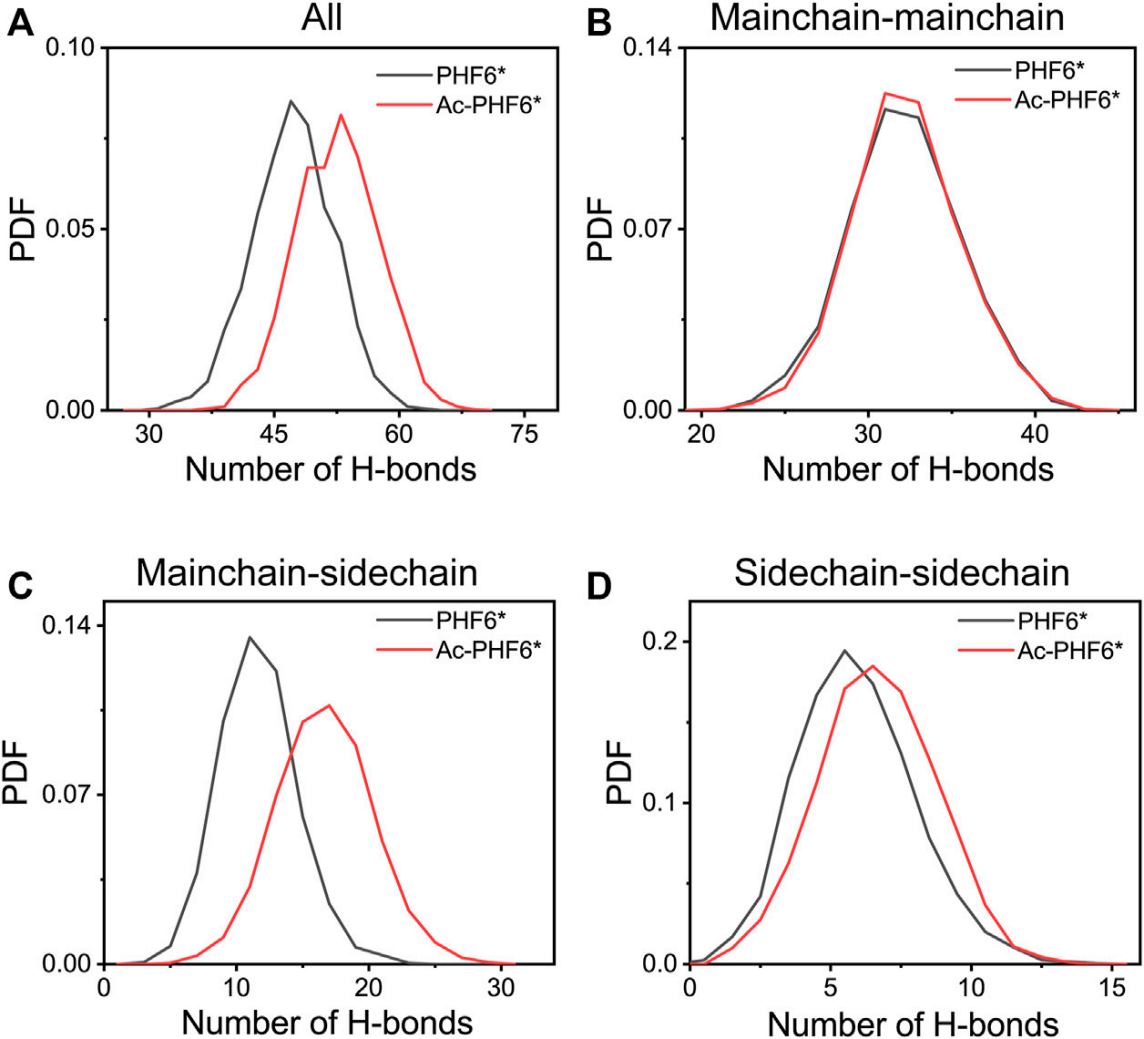
Probability distribution function of total **(A)**, mainchain-mainchain **(B)**, mainchain-sidechain **(C)** and sidechain-sidechain **(D)** inter-chain H-bond number in the PHF6* and Ac-PHF6* systems.

In order to explore the underlying mechanism by which K280 acetylation has a higher probability to form β-sheets, we calculated the PDF of the number of inter-chain H-bonds in two systems. As seen in Figure 5A, K280 acetylation significantly enhances the total number of H-bonds, leading to the peak value shifting from 47 to 53. Figures 5B–D presents the distribution of mainchain-mainchain (MC-MC), mainchain-sidechain (MC-SC) and sidechain-sidechain (SC-SC) H-bond number. For the inter-chain mainchain H-bond (Figure 5B), a peak centered at 31 is observed in PHF6* system, while this peak becomes higher in Ac-PHF6* system, implying that K280 acetylation strengthens the formation of inter-chain mainchain H-bonds to a degree. Figures 5C,D shows that the increased H-bonds also appear in the MC-SC and SC-SC of PHF6* oligomer. Given the crucial role of H-bonds in the β-sheet formation and oligomerization of Tau protein, the above results indicate that K280 acetylation favors the H-bonds formation of PHF6* peptides, thus accelerating the aggregation of PHF6*.

**FIGURE 6:**
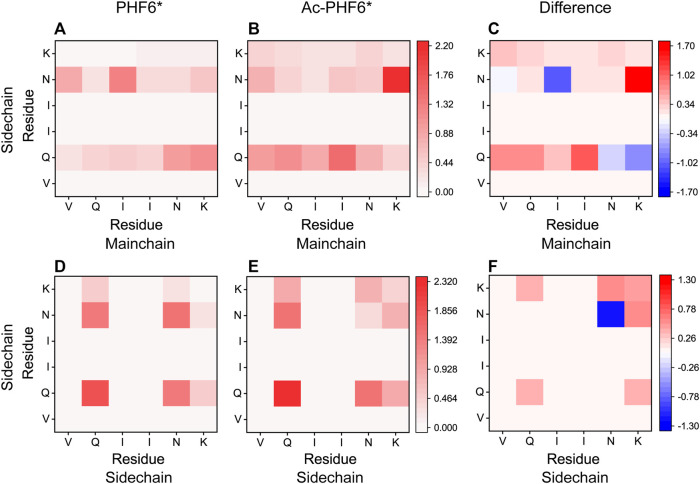
Analyses of K280 acetylation on the H-bond interactions: formation maps of MC-SC and SC-SC H-bonds in the PHF6* **(A,D)** and Ac-PHF6* **(B,E)** systems. The H-bond map for the difference between two systems is given in **(C,F)**. The differences are calculated using the H-bond number of residue pairs in Ac-PHF6* system minus those in PHF6* system. The number of H-bond is averaged over twelve peptide chains.

We then plotted residue-residue H-bonds formation maps in the PHF6* and Ac-PHF6* systems to further investigate the effect of K280 acetylation on the H-bond interactions. As shown in Figure 6A, in the PHF6* system, the sidechain of Q276 and N279 forms more H-bonds with the mainchain of N279/K280 and V275/I277, respectively. K280 acetylation affects the MC-SC H-bond map and increases the number of most of these MC-SC H-bonds. This phenomenon can be clearly seen in the H-bond formation map for the difference between two systems in Figure 6C. The number of MC-SC H-bond formed by residue pairs N279 (sidechain)-K280 (mainchain) is remarkable increased after acetylation, and the increment is far higher than that in other residue pairs. We further calculated the percentage occupation of H-bonds formed by sidechain of N279 and mainchain of K280 in two systems. Table 1 shows that in PHF6* system, K280 serves as a hydrogen acceptor (K280 O) of N279 (N279 ND2) with an occupied percentage of 15.34% and as a hydrogen donor (K280 N) to N279 (N279 OD1) in more than 22% of the simulation. When K280 is acetylated, the occupied percentages of the two H-bond pairs increase to 41.88 and 73.76%, respectively. For the SC-SC H-bond map (Figures 6D–F), we found that H-bond number of four residue pairs (N279-K280, K280-K280, Q276-K280 and Q276-Q276) are obviously increased after acetylation. Among them, the N279-K280 displays a highest increment, showing the same result as that in the MC-SC H-bond map. We also presented the profiles of SC-SC H-bonds formed by N279 and K280 in two systems in Table 1. A same H-bond pair is formed between the oxygen atom (OD1) of N279 and the nitrogen atom (NZ) of K280 in the PHF6* and Ac-PHF6* systems, with the occupied percentage of 9.9 and 18.24% respectively. Besides, acetylated K280 also acts as a hydrogen acceptor (K280 OI2) of N279 (N279 ND2) via its acetyl group in a consistent manner (over 32% incidence), reflecting the important role of the acetyl group of acetylated K280 in the H-bond formation. The positive role of modified group in the formation of H-bonds was also reported in a recent study. Liu et al. investigated the effect of phosphorylation on the conformational features of Tau_192-212_, and found the oxygen atoms on the phosphate group are involved in the formation of multiple key H-bonds that making the structure more stable (Liu et al., 2021). In addition, we plotted the MC-MC H-bond map in Supplementary Figure S5, and found that after acetylation, the MC-MC H-bond numbers of residue pairs involving residues N279/K280 are obviously increased. These results indicate that acetylation of K280 significantly alters the H-bond formation network, and enhances the peptide-peptide interaction.

**TABLE 1:** Calculated percentage occupation (%) of the MC-SC and SC-SC H-bonds formed by N279 and K280 in PHF6* and Ac-PHF6* systems.

H-bonds	PHF6* system			Ac-PHF6* system		
	Acceptor	Donor	Occupied percentage (%)	Acceptor	Donor	Occupied percentage (%)
MC-SC	K280@ON279@OD1	N279@ND2K280@N	15.3422.34	K280@O	N279@ND2	41.88
				N279@OD1	K280@N	73.76
SC-SC	N279@OD1	K280@NZ	9.9	N279@OD1	K280@NZ	18.24
				K280@OI2	N279@ND2	32.78

**FIGURE 7:**
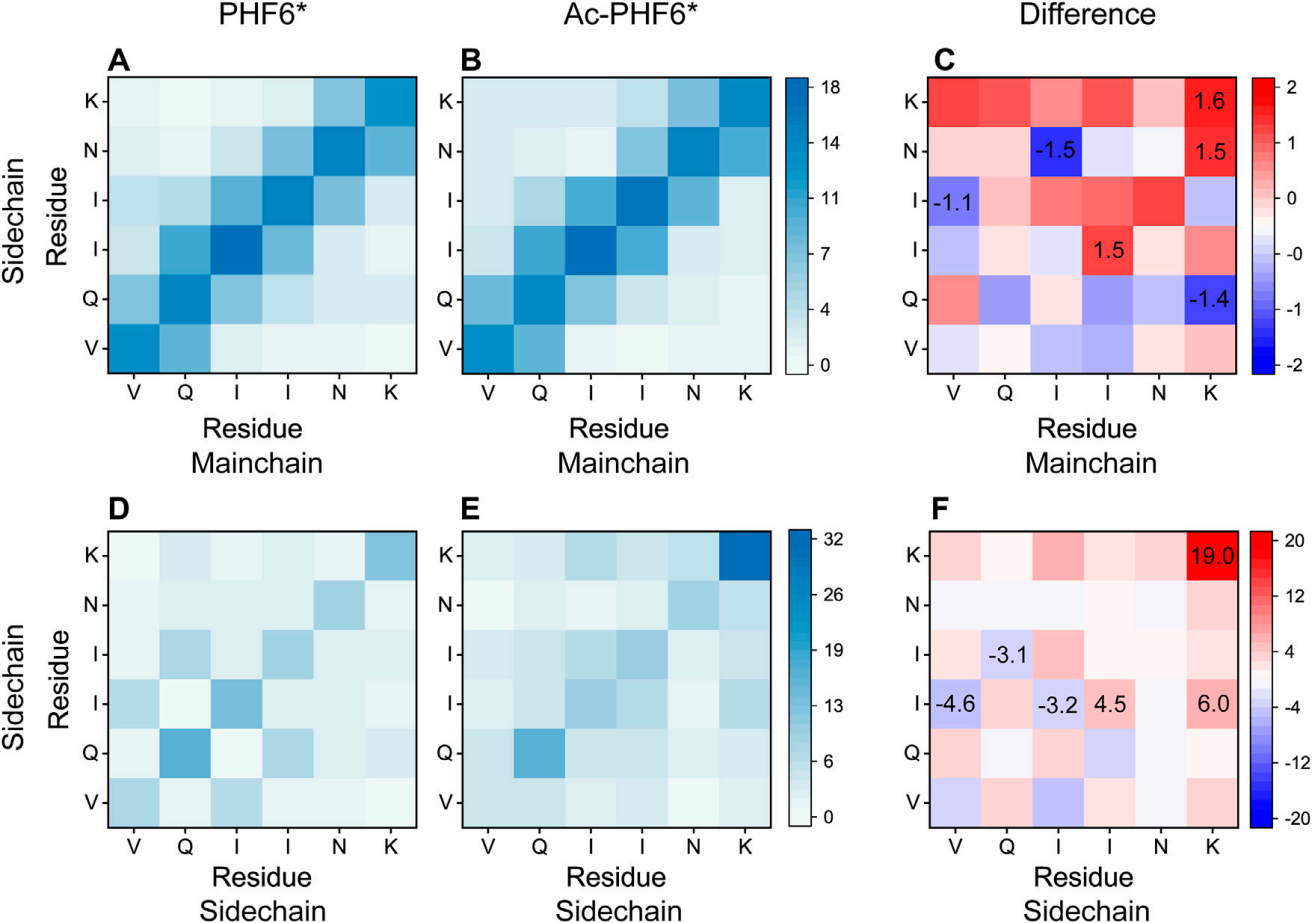
Analyses of the effect of acetylation on MC-SC and SC-SC contact number maps for PHF6* oligomer in the PHF6* **(A,D)** and Ac-PHF6* **(B,E)** systems. MC-SC **(C)** and SC-SC **(F)** contact number maps for the difference between PHF6* and Ac-PHF6* systems. The contact number is normalized with the total peptide chain number of 12 in each system.

To explore the effect of K280 acetylation on pairwise residue-residue interactions of PHF6* oligomers, we plotted the MC-SC and SC-SC contact number maps in the PHF6* and Ac-PHF6* systems. As seen in Figure 7, the contact number maps in two systems display distinct MC-SC and SC-SC interaction patterns, implying that residue-residue interactions are remarkably altered by K280 acetylation. The relative higher contact numbers along the right diagonal of the contact maps shown in (Figures 7A,D) and Supplementary Figure S6A demonstrate that peptide chains in PHF6* system mainly adopt parallel alignments. When K280 is acetylated (Figures 7B,E and Supplementary Figure S6B), contact numbers of most residue pairs along the right diagonal of the maps are increased, suggesting that K280 acetylation enhances interactions of these residue pairs responsible for the parallel β-sheet formation. The MC-SC and SC-SC contact number maps for the difference between two systems are presented in Figures 7C,F. The differences are calculated using the contact number of residue pairs in Ac-PHF6* system minus those in PHF6* system. K280-K280, K280-N279 and K280-I277 pairs display a high MC-SC and SC-SC contact number difference, showing acetylation strengthens the interactions of K280 with the hydrophobic residues of PHF6*. This phenomenon may be attributed to the extra hydrophobic group acetyl (CH3CO) of K280 after acetylation, leading an enhanced hydrophobic interaction between K280 and other hydrophobic residues. It has been demonstrated that hydrophobic interaction is closely associated with the formation and stabilization of Tau aggregates (Dyson et al., 2006; Lin et al., 2021). These results indicate that the enhanced hydrophobicity induced by K280 acetylation could strengthen the MC-SC and SC-SC interactions that are critical for PHF6* oligomerization and fibrillization. Meanwhile, we found that SC-SC contact numbers in two systems are much higher than the MC-SC contact numbers, and K280 acetylation leads to a higher increment of SC-SC contact numbers than that of MC-SC, revealing SC-SC interaction plays an important role in the formation of PHF6* oligomers.

## Conclusion

In summary, we have investigated the self-assembly of the fibril-nucleating core motif PHF6* of Tau protein and the effect of K280 acetylation on PHF6* oligomerization, by performing two 400 ns atomistic REMD simulations starting from a random state. Our simulations show that K280 acetylation markedly increases the β-sheet probability of PHF6* and enhances the formation of larger β-sheet-rich oligomers. The conformations in the PHF6* system display a high diversity, while K280 acetylation reduces the structural diversity and self-assemblies into some ordered β-barrels and bilayer β-sheets. The analyses of SASA and end-to-end distance show that after K280 acetylation, PHF6* peptides assemble into a more compact state and have a larger propensity to adopt extended β-sheet conformations. Moreover, we found that the acetylation of K280 significantly alters the H-bond and contact network and strengthens the residue-residue interactions, especially at the acetylation site. Overall, this study reveals the mechanisms that K280 acetylation affects Tau aggregation at the atomic level, and may provide valuable information for developing a PTM-modifying therapeutic strategy for tauopathies.

## Data Availability

The raw data supporting the conclusion of this article will be made available by the authors, without undue reservation.
