# Supplementary figures and images for: Unraveling the Influence of K280 Acetylation on the Conformational Features of Tau Core Fragment: A Molecular Dynamics Simulation Study
Source: Front Mol Biosci. 2021 Dec 13;8:801577. doi: 10.3389/fmolb.2021.801577 (PMC8710698; doi:10.3389/fmolb.2021.801577)

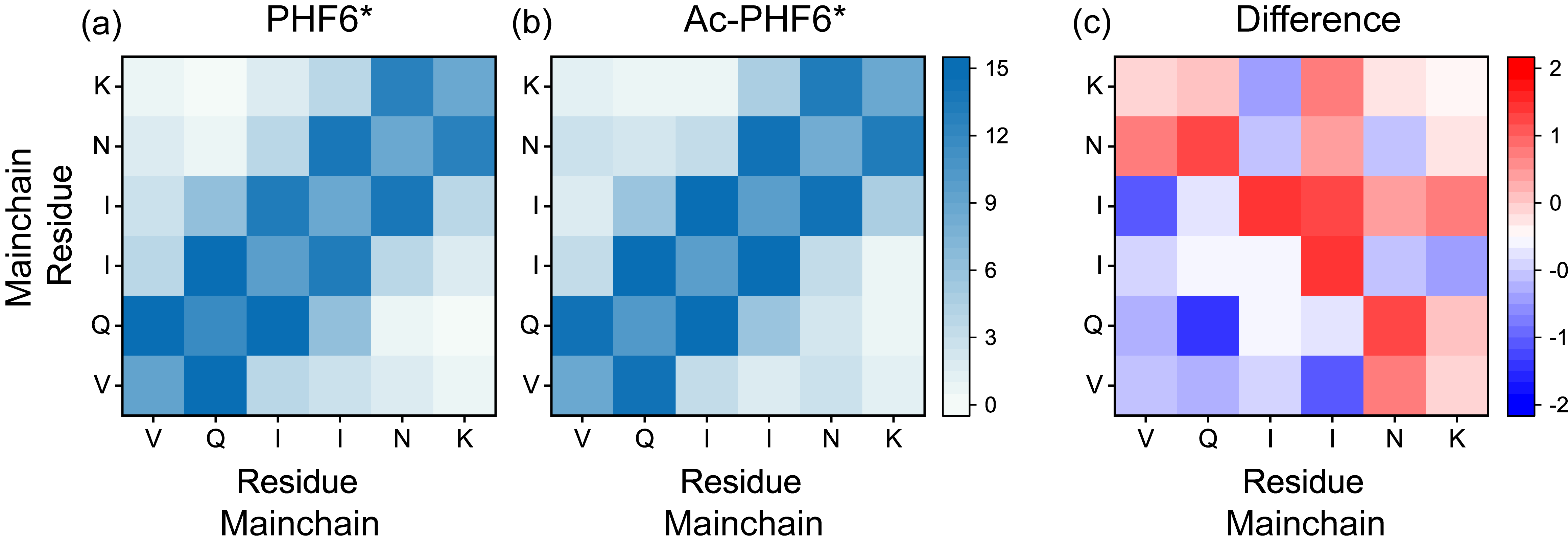

Supplement: Supplementary file 1 [file Image6.TIF]

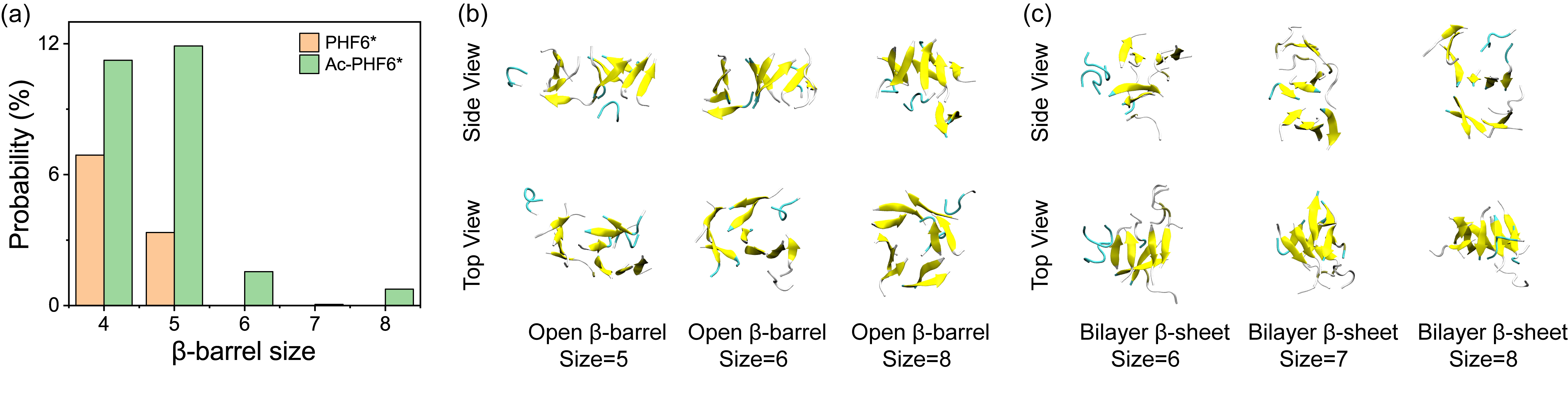

Supplement: Supplementary file 2 [file Image3.TIF]

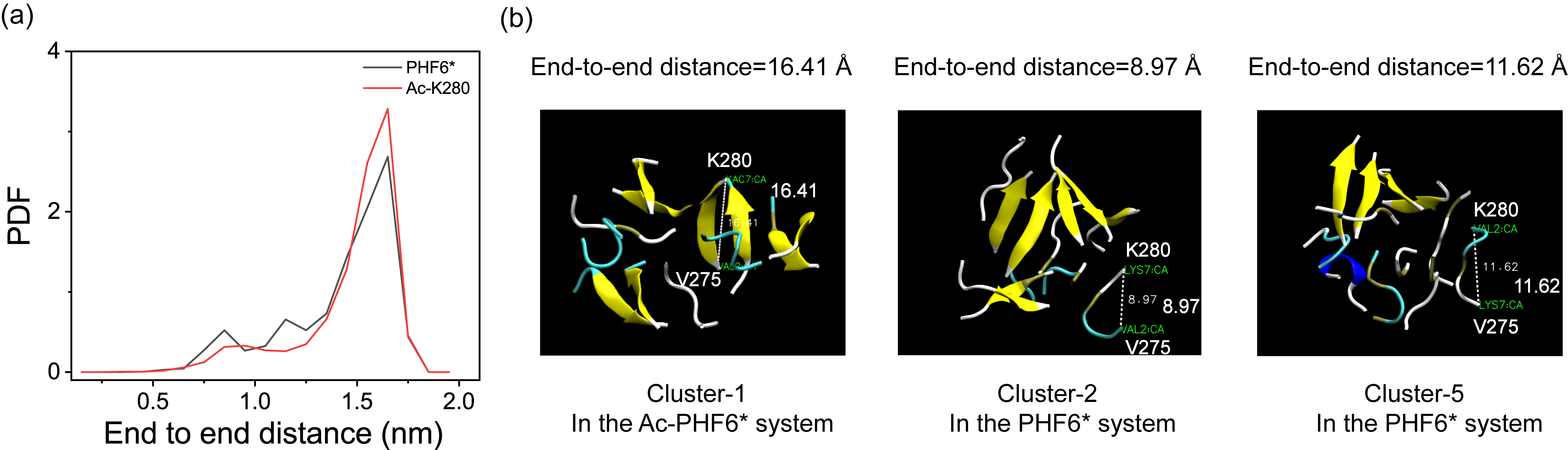

Supplement: Supplementary file 3 [file Image4.TIF]

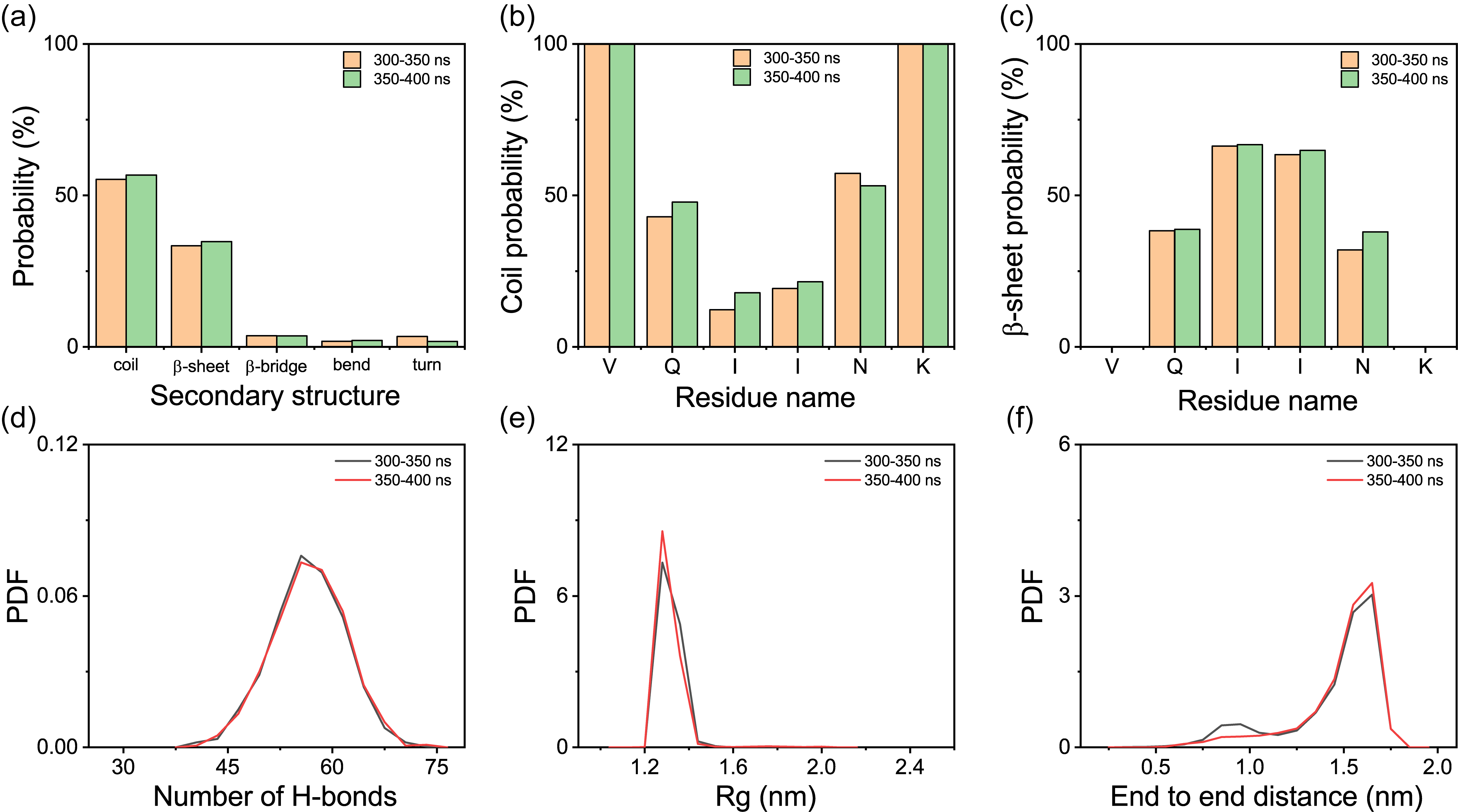

Supplement: Supplementary file 5 [file Image2.TIF]

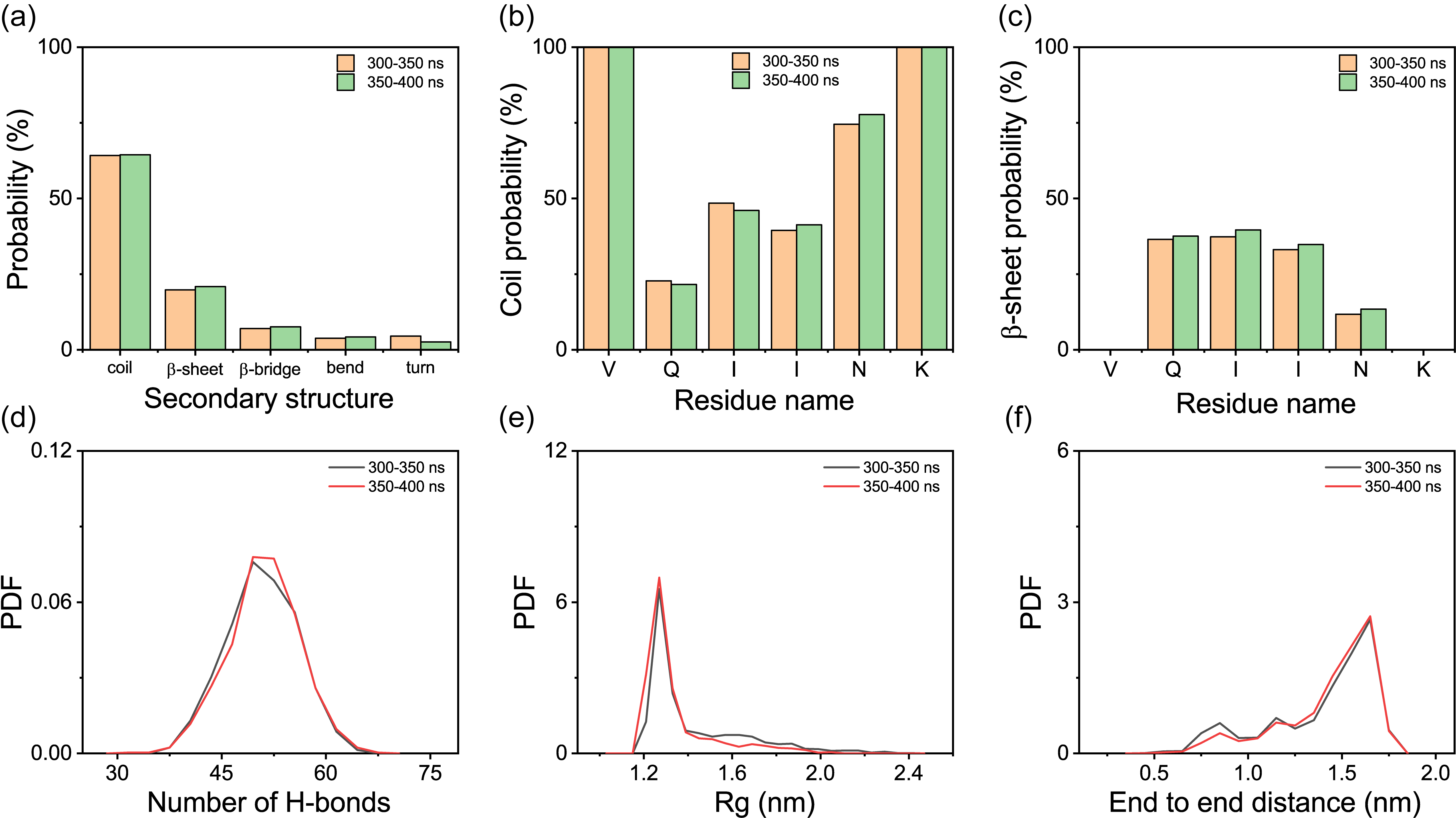

Supplement: Supplementary file 6 [file Image1.TIF]

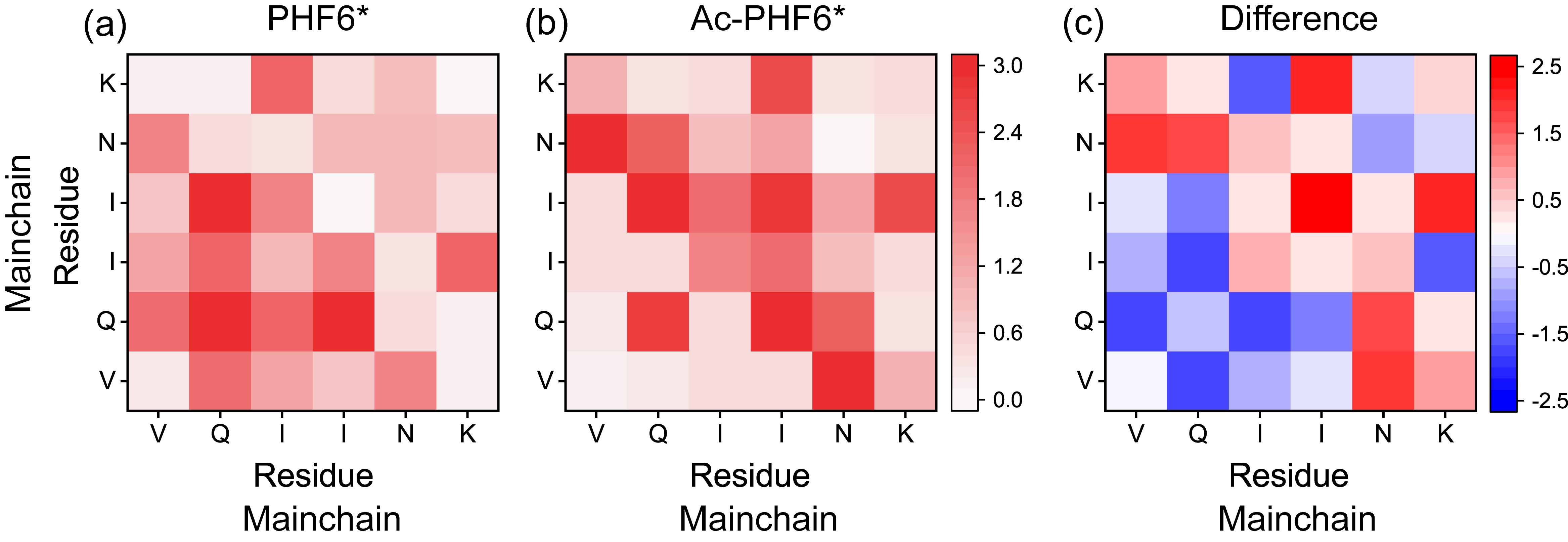

Supplement: Supplementary file 7 [file Image5.TIF]
