# Supplementary material for: Unraveling the Influence of K280 Acetylation on the Conformational Features of Tau Core Fragment: A Molecular Dynamics Simulation Study
Source: Front Mol Biosci. 2021 Dec 13;8:801577. doi: 10.3389/fmolb.2021.801577 (PMC8710698; doi:10.3389/fmolb.2021.801577)
Supplement: Supplementary file 4 [file DataSheet1.doc]

**Supplementary Material**

This material contains the convergence check of REMD simulations, and temperature list of REMD simulation, six supplementary figures (Fig. S1-S6) and one supplementary table (Tab. S1).

**Six supplementary figures**


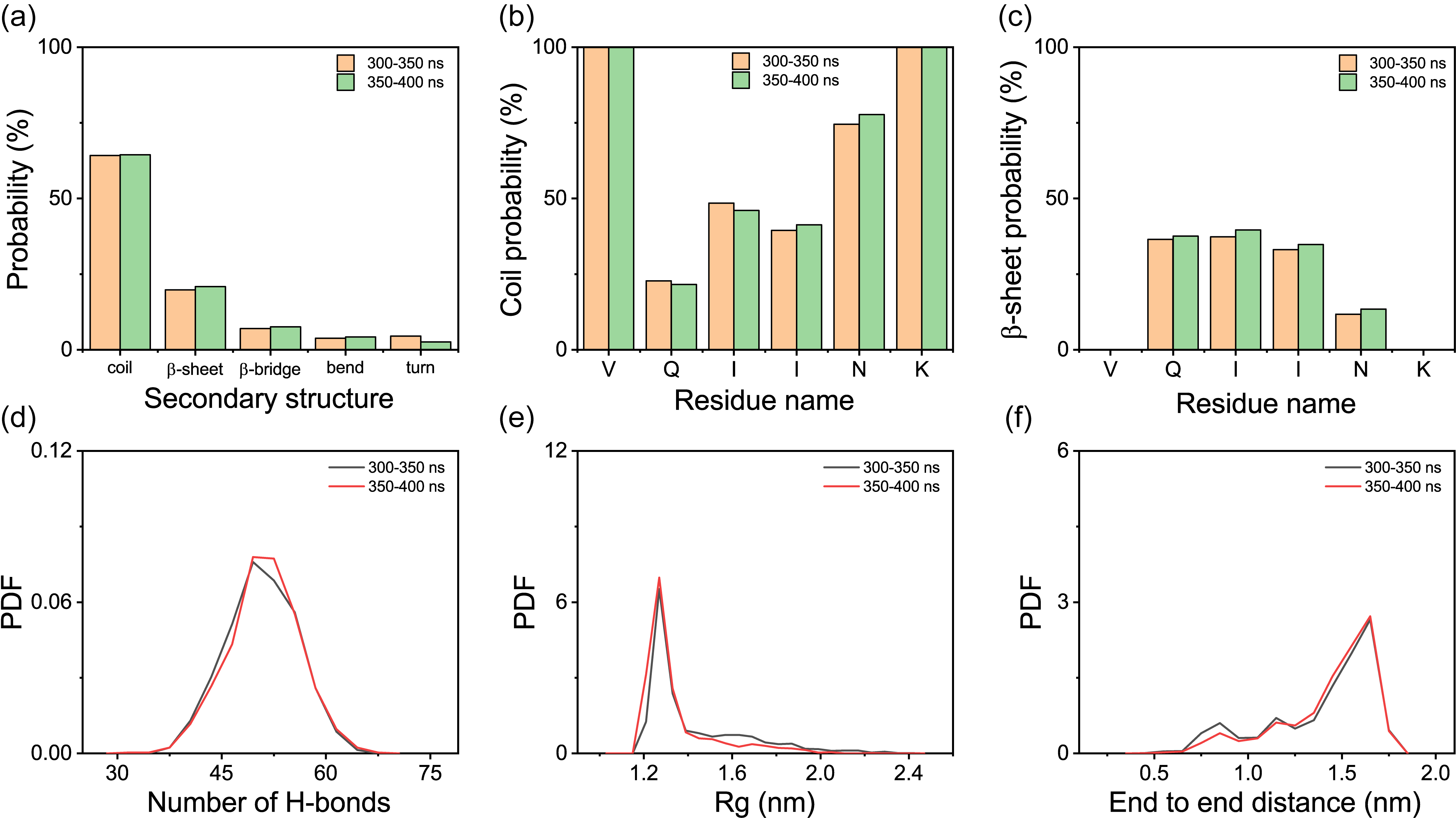


**FIGURE S1** **|** Probability of each type of secondary structure (including coil, β-sheet, β-bridge, bend and turn) averaged over all residues **(a)**, coil **(b)** and β-sheet **(c)** probability as a function of amino acid residue, probability distribution function of the number of H-bond **(d)**, Rg **(e)** and end-to-end distance **(f)** of PHF6* oligomers in the PHF6* system using two different time intervals (300-350 ns and 350-400 ns).


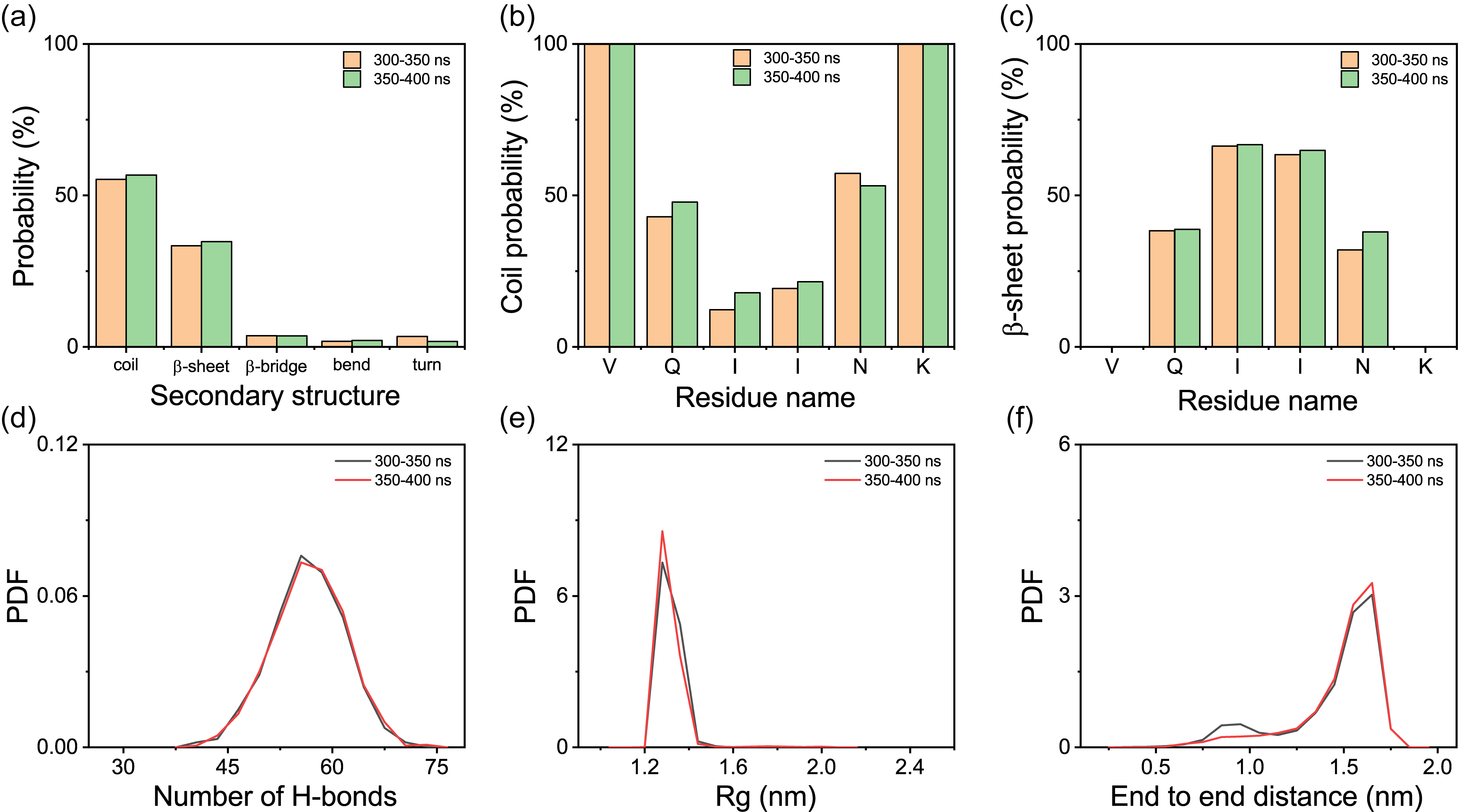


**FIGURE S2** **|** Probability of each type of secondary structure (including coil, β-sheet, β-bridge, bend and turn) averaged over all residues **(a)**, coil **(b)** and β-sheet **(c)** probability as a function of amino acid residue, probability distribution function of the number of H-bond **(d)**, Rg **(e)** and end-to-end distance **(f)** of PHF6* oligomers in the Ac-PHF6* system using two different time intervals (300-350 ns and 350-400 ns).


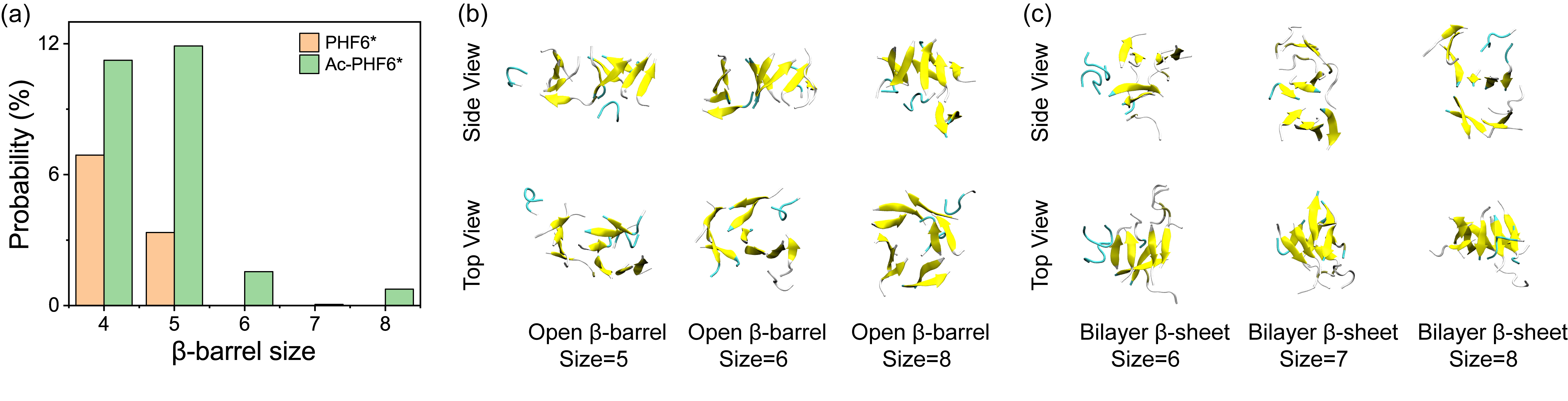


**FIGURE S3** **|** Probability of different sizes of β-barrel in PHF6* and Ac-PHF6* systems **(a)**. Representative PHF6* oligomers in the Ac-PHF6* system containing different sizes of ordered β-barrels **(b)** and bilayer β-sheets **(c)**. The different sizes of β-barrel or bilayer β-sheet are denoted by the number of β-strands forming the barrel or bilayer β-sheet. The structures are shown in two different views: top view and side view.


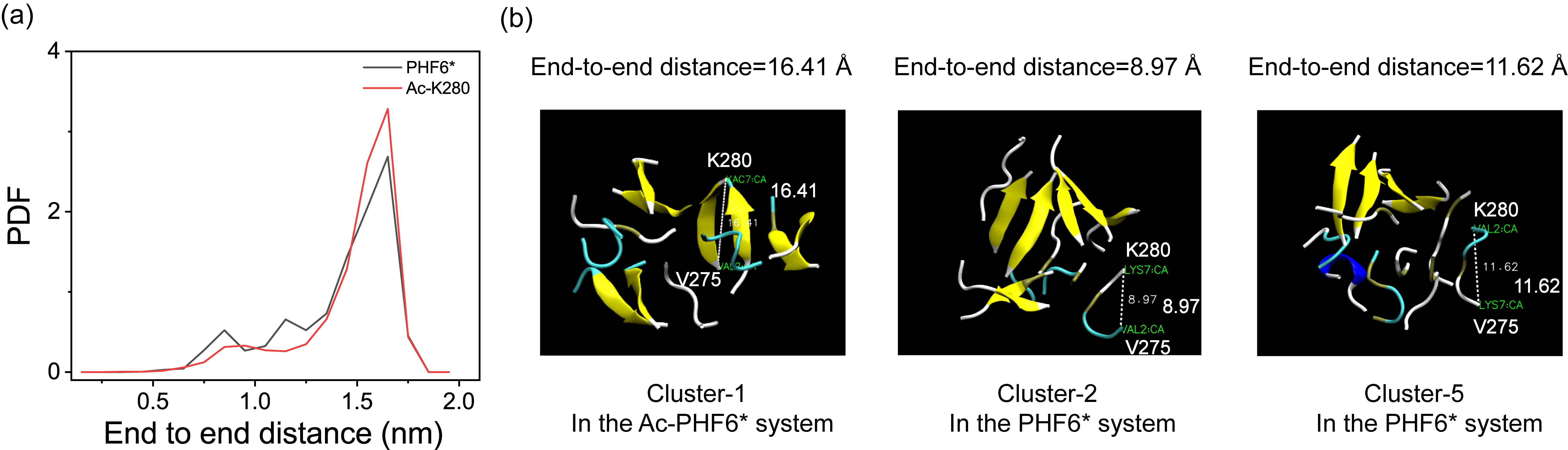


**FIGURE S4** **|** Analyses of end-to-end distance of each peptide chain in PHF6* and Ac-K280 systems **(a)**. Snapshots of PHF6* peptide with an end-to-end distance of 16.41 Å, 8.97 Å and 11.62 Å **(b)**. The end-to-end distance is calculated from the V275 Cα atom to the K280 Cα atom.


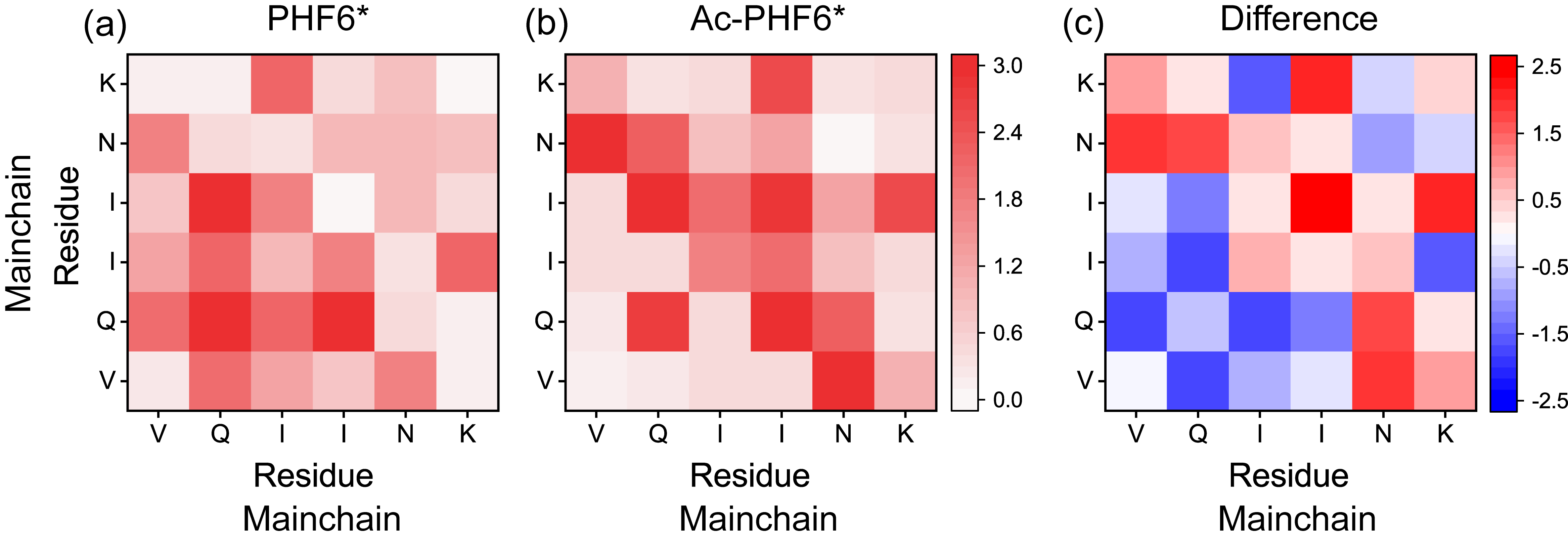


**FIGURE S5** **|** Analyses of K280 acetylation on the H-bond interactions: formation maps of MC-MC H-bonds in the PHF6* **(a)** and Ac-PHF6* **(b)** systems. The H-bond map for the difference between two systems is given in **(c)**. The differences are calculated using the H-bond number of residue pairs in Ac-PHF6* system minus those in PHF6* system. The number of H-bond is averaged over twelve peptide chains.


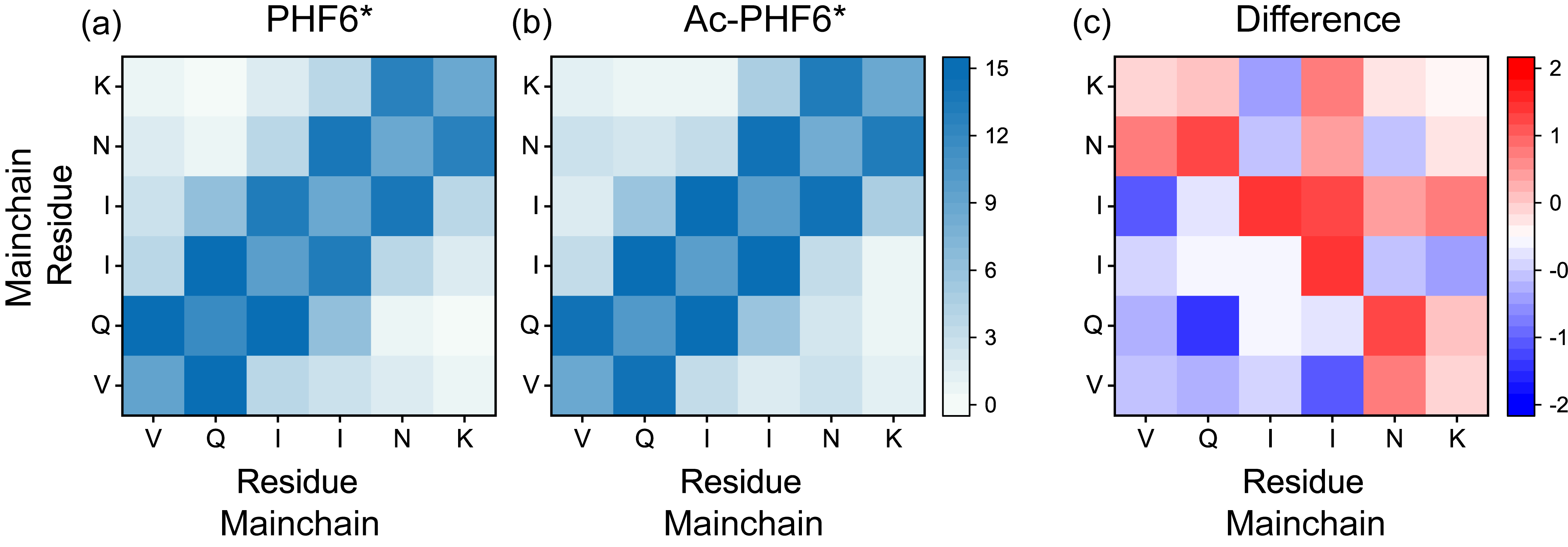


**FIGURE S6** **|** Analyses of the effect of acetylation on MC-MC contact number maps for PHF6* oligomer in the PHF6* **(a)** and Ac-PHF6* **(b)** systems. MC-MC **(c)** contact number maps for the difference between PHF6* and Ac-PHF6* systems. The contact number is normalized with the total peptide chain number of 12 in each system.

**SUPPLEMENTARY TABLE 1** **|** Temperature lists of REMD simulation for PHF6* and Ac-PHF6* systems.

| 308.00 | 310.01 | 312.04 | 314.07 | 316.11 | 318.16 | 320.26 | 322.33 |
| --- | --- | --- | --- | --- | --- | --- | --- |
| 324.41 | 326.52 | 328.63 | 330.74 | 332.93 | 335.06 | 337.21 | 339.37 |
| 341.54 | 343.72 | 345.91 | 348.11 | 350.32 | 352.54 | 354.77 | 357.02 |
| 359.27 | 361.54 | 363.82 | 366.11 | 368.41 | 370.73 | 373.05 | 375.39 |
| 377.74 | 380.10 | 382.47 | 384.86 | 387.25 | 389.66 | 392.08 | 394.51 |
| 396.96 | 399.41 | 401.88 | 404.37 | 406.86 | 409.36 | 411.89 | 414.00 |
